# Statistical Validation of Risk Alleles in Genetic Addiction Risk Severity (GARS) Test: Early Identification of Risk for Alcohol Use Disorder (AUD) in 74,566 Case–Control Subjects

**DOI:** 10.3390/jpm12091385

**Published:** 2022-08-26

**Authors:** Kenneth Blum, David Han, Ashim Gupta, David Baron, Eric R. Braverman, Catherine A. Dennen, Shan Kazmi, Luis Llanos-Gomez, Rajendra D. Badgaiyan, Igor Elman, Panayotis K. Thanos, Bill W. Downs, Debasis Bagchi, Marjorie C. Gondre-Lewis, Mark S. Gold, Abdalla Bowirrat

**Affiliations:** 1 Graduate College, Western University Health Sciences, Pomona, CA 91766, USA; 2 Institute of Psychology, ELTE Eötvös Loránd University, Egyetem tér 1-3, 1053 Budapest, Hungary; 3 The Kenneth Blum Institute on Behavior & Neurogenetics, LLC., Austin, TX 78701, USA; 4 Department of Psychiatry, School of Medicine, University of Vermont, Burlington, VT 05405, USA; 5 Dayton VA Medical Centre, Department of Psychiatry, Boonshoft School of Medicine, Wright State University, Dayton, OH 45324, USA; 6 Division of Precision Nutrition, Victory Nutrition International, LLC., Lederoch, PA 19438, USA; 7 Department of Management Science and Statistics, University of Texas at San Antonio, San Antonio, TX 78249, USA; 8 Future Biologics, Lawrenceville, GA 30043, USA; 9 Department of Family Medicine, Jefferson Health Northeast, Philadelphia, PA 19114, USA; 10 Department of Psychiatry, South Texas Veteran Health Care System, Audie L. Murphy Memorial VA Hospital, Long School of Medicine, University of Texas Medical Center, San Antonio, TX 78229, USA; 11 Center for Pain and the Brain (P.A.I.N Group), Department of Anesthesiology, Critical Care & Pain Medicine, Boston Children’s Hospital, Boston, MA 02115, USA; 12 Cambridge Health Alliance, Harvard Medical School, Cambridge, MA 02139, USA; 13 Behavioral Neuropharmacology and Neuroimaging Laboratory, Department of Pharmacology and Toxicology, Jacobs School of Medicine and Biomedical Sciences, Clinical Research Institute on Addictions, University at Buffalo, Buffalo, NY 14203, USA; 14 Department of Psychology, University at Buffalo, Buffalo, NY 14260, USA; 15 Department of Pharmaceutical Science, College of Pharmacy & Health Sciences, Texas Southern University, Houston, TX 77004, USA; 16 Department of Psychiatry and Behavioral Sciences, Howard University College of Medicine, Washington, DC 20059, USA; 17 Department of Psychiatry, Washington University School of Medicine, St. Louis, MO 63110, USA; 18 Department of Molecular Biology, Adelson School of Medicine, Ariel University, Ariel 40700, Israel

**Keywords:** statistical validation of GARS, neurotransmitters, dopamine, opioids, odds ratios, Genetic Addiction Risk Severity (GARS), Reward Deficiency Syndrome (RDS)

## Abstract

Since 1990, when our laboratory published the association of the DRD2 Taq A1 allele and severe alcoholism in *JAMA*, there has been an explosion of genetic candidate association studies, including GWAS. To develop an accurate test to help identify those at risk for at least Alcohol Use Disorder (AUD), Blum’s group developed the Genetic Addiction Risk Severity (GARS) test, consisting of ten genes and eleven associated risk alleles. In order to statistically validate the selection of these risk alleles measured by GARS, we applied strict analysis to studies that investigated the association of each polymorphism with AUD or AUD-related conditions published from 1990 until 2021. This analysis calculated the Hardy–Weinberg Equilibrium of each polymorphism in cases and controls. If available, the Pearson’s *χ*^2^ test or Fisher’s exact test was applied to comparisons of the gender, genotype, and allele distribution. The statistical analyses found the OR, 95% CI for OR, and a post-risk for 8% estimation of the population’s alcoholism prevalence revealed a significant detection. The OR results showed significance for DRD2, DRD3, DRD4, DAT1, COMT, OPRM1, and 5HTT at 5%. While most of the research related to GARS is derived from our laboratory, we are encouraging more independent research to confirm our findings.

## 1. Introduction

Substance Use Disorder (SUD) clinicians and scientists face great obstacles in the fight against the current Alcohol Use Disorder (AUD) and opioid pandemic worldwide. Although significant progress has been made, the death toll from narcotic overdoses reached 72,000 fatalities in the United States alone in 2017. The National Institute on Alcohol Abuse and Alcoholism (NIAAA) and the National Institute on Drug Abuse (NIDA) have not been able to adequately address the severity of the current substance abuse epidemic using novel Food and Drug Administration (FDA) approved treatments. Medication-Assisted Treatments (MAT) that have been approved by the FDA primarily work by modulating the function of the nucleus accumbens. This is accomplished by inhibiting dopamine release and function at the pre-neuron of the nucleus accumbens [1,2]. Despite the success of MAT in reducing overdose deaths, costs, and health care events, a long-term strategy to return MAT patients to premorbid functioning is necessary. Unfortunately, MAT fail routinely [3], and upon discontinuation, the rate of overdose and relapse among treated patients mirrors that of untreated patients. In addition, MAT may induce persistent neurological changes that compromise endorphin, dopamine, and multiple brain systems. While the chronic use of agonist therapies may be necessary in the absence of other options, we caution that data on chronic vs. acute use harm reduction is lacking [4,5]. However, there is evidence that the long-term use of agonist treatments themselves, such as those used for Opioid Use Disorder (OUD), may in fact precipitate Reward Deficiency Syndrome (RDS) [5], and result in all the negative sequalae associated with it.

Drug overdoses are the most prevalent in the United States, but they are an international problem that demands urgent, innovative solutions. Short-term opioid substitution therapy can reduce harm; however, long-term patients risk being locked into a lifetime of SUD [5]. Alternatively, inducing “psychological extinction” by weakening a conditioned response over time using the narcotic antagonist, naltrexone, blocks delta and mu opioid receptors [6]. However, one difficulty encountered when using narcotic antagonism is compliance, which is moderated by the individual’s genetic antecedents [7]. Other FDA-approved treatments for alcoholism function through the inhibition of dopaminergic signaling [8,9].

There is increasing movement to opt for the non-addicting narcotic antagonist naltrexone to treat AUD. Recent studies have shown that naltrexone is beneficial by attenuating craving via “psychological extinction” and reducing relapse. Buprenorphine is currently the MAT of choice, but injectable naltrexone plus an agent to improve dopaminergic function and tone may renew interest amongst addiction physicians and patients. Even with the extended injectable option, there is still poor compliance. As such, our group described an open-label investigation in humans showing improvement in naltrexone compliance and outcomes with dopamine augmentation with the pro-dopamine regulator KB220 (262 days) compared to naltrexone alone (37 days) [6]. This well-studied complex consists of amino-acid neurotransmitter precursors and enkephalinase inhibitor therapy compared to standard treatment. Consideration of this novel paradigm shift may assist in addressing the current opioid and alcohol epidemics and the broader question of reward deficiency in general.

Blum’s group first coined the term “RDS” in 1995 to identify a group of behaviors with a common neurobiological mechanism associated with a polymorphic allelic propensity for hypodopaminergia as determined by measuring a number of reward genes and associated risk alleles. Understanding this, coupled with the rising scientific consensus regarding the underlying concept of RDS, supports the hypothesis of a common mechanism that groups both chemical and behavioral addictions together [10]. The disruption of neuromodulation and its negative impact on neurotransmission resulting from chronic exposure to drugs and behavioral addictions requires an approach that involves attaining “dopamine homeostasis,” especially for AUD [11].

Subsequent large-scale genomics studies have had limited success in identifying alleles implicated in addiction and RDS. Despite the utility of Genome-Wide Association Studies (GWAS) and next-generation sequencing, some key issues exist. For example, GWAS is a useful tool for identifying new clusters of genes that may contribute to genetic antecedents related to specific RDS behaviors such as AUD. The next key step following the GWAS results is to converge the data to identify individual candidate genes. Thus, if there is indeed a blueprint or clue regarding specific known genes and associated polymorphic risk alleles linked to a specific phenotype such as AUD, it is significant even though the contribution of each gene may be small.

**Figure 1:**
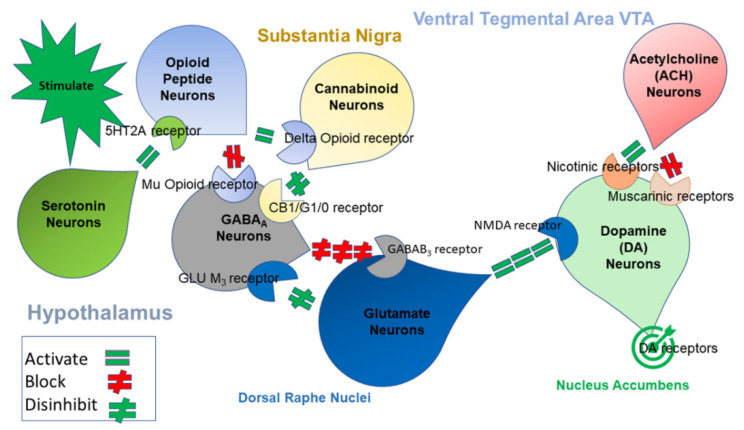
Illustrates the interaction of at least six major neurotransmitter pathways involved in the Brain Reward Cascade (BRC). In the hypothalamus, environmental stimulation causes the release of serotonin, which in turn, via 5HT-2a receptors, activates (the green, equal sign) the subsequent release of opioid peptides into the hypothalamus. Then, the opioid peptides have two distinct effects, possibly via two different opioid receptors. (A) Inhibits (the red hash sign) through the Mu-opioid receptor (possibly via enkephalin) and projects to the substania nigra to GABAA neurons. (B) Stimulates (the green, equal sign) cannabinoid neurons (e.g., anandamide and 2-archydonoglcerol) through beta–endorphin-linked delta receptors, which in turn inhibit GABAA neurons at the substania nigra. Cannabinoids, primarily 2-archydonoglcerol, when activated, can also indirectly disinhibit (the red hash sign) GABAA neurons in the substania nigra through activation of G1/0 coupled to CB1 receptors. Similarly, glutamate neurons located in the Dorsal Raphe Nuclei (DRN) can indirectly disinhibit GABAA neurons in the substania nigra by activating GLU M3 receptors (the red hash sign). GABAA neurons, when stimulated, will, in turn, powerfully (the red hash signs) inhibit Ventral Tegmental Area (VTA) glutaminergic drive via GABAB 3 neurons. Finally, glutamate neurons in the VTA will project to dopamine neurons through NMDA receptors (the green, equal sign) to preferentially release dopamine at the NAc, shown as a bullseye indicating well-being (Blum et al. with permission).

Several neurotransmitters are involved in the processing of reward and punishment. These pathways involve at least six quintessential neurotransmitters and many second messengers linked to the mesolimbic and Pre-Frontal Cortex (PFC). One function is to regulate the final pathway of “wanting”, causing net neuronal dopamine release. Figure 1 provides a schematic representation of the Brain Reward Cascade (BRC), showing the interaction of serotonergic, cannabinoidergic, opioidergic, GABAergic, glutaminergic, and dopaminergic systems related to net dopamine release at the Nucleus Accumbens (NAc). In this article, the authors highlight dopamine based on the understanding that the healthy processing of an initial action potential in the brain requires the integrity of the entire neurotransmitter complex of the brain reward circuitry. The cascading interactions result in the balanced release of dopamine at the NAc and across many brain regions. These regions are involved in cognition (memory), motivation, stress reduction, decision-making, recall, pleasure, cravings, drug reinstatement, and well-being.

In keeping with the current literature, we are cognizant of the many second messengers as well as the interrelatedness of at least seven finite neurotransmitter systems involving dopaminergic activity. We are not proposing that these genes are unimportant; rather, that they influence the final dopaminergic pathway and its function at the nucleus accumbens. We make this distinction to avoid confusion and to inform the readership that hypodopaminergia is, in fact, a significant factor that influences unwanted substance and non-substance addictive behavior seeking. In this regard, being cognizant of these difficulties and awaiting further research, the BRC was utilized as a blueprint (Figure 1) for a literature review to determine each allele associated with a hypodopaminergic polymorphism. These alleles were proposed for a GARS panel in case–control studies, specifically for alcoholism (Table 1).

Blum’s laboratory worked toward developing an accurate genetic test to predict the liability risk for RDS behaviors, including AUD [12,13]. Geneus Health LLC scientists, in conjunction with their Genomic Testing Centre, successfully developed the first Genetic Addiction Risk Severity (GARS) test. The actual association to determine risk using a clinical outcome, the Addiction Severity Index Media Version (ASI-MV), was accomplished by the Institute of Behavioral Genetics, University of Colorado, Boulder [14,15,16,17,18,19,20,21].

The development of this patented GARS test involved the selection of ten reward candidate genes that reflected a hypodopaminergic trait, including the dopamine receptors (DRD1, 2, 3, 4); Dopamine Transporter (DAT1); serotonin transporter, COMT, MAO, GABA, Mu opiate receptor, Single Nucleotide Polymorphisms (SNPs), and point mutations. The genes determined to negatively influence the net release of dopamine at the brain reward site were chosen from thousands of association studies providing clear evidence of specific risk for all addictions.

The idea of “dopamine homeostasis” stems from the basic understanding of the importance of having a “normal” balance of not only dopamine but many well-characterized neurotransmitters and even metabolic systems. To that end, further research is necessary to understand the normative interface between hedonostatic and homeostatic mechanisms and how their collapse is manifested in the mounting allostatic load and eventual addiction. One additional thought worthwhile to consider herein is an ensuing field of addiction diabetology that could propel a unique multi-disciplinary collaboration unrestricted by unnecessary boundaries separating addiction medicine and psychiatry from diabetology and its related disciplines (e.g., dietology, primary care, nephrology, and ophthalmology), thus contributing to a greater depth and sophistication of clinical formulations and therapeutic interventions.

This article aimed to provide the field with statistical validation of a number of select reward-based genes and associated polymorphisms that primarily induce reduced dopamine function in the meso-limbic brain reward circuitry. While the genetics proposed herein are based on AUD cases and controls, we encourage further work to confirm this and other known “reward processing dysregulation” as an expanded phenotype.

## 2. Materials and Methods

### 2.1. Literature Search and Inclusion of Eligible Studies

Studies investigating the association of each polymorphism with AUD or AUD-related conditions were provided for a meta-analysis. They were published from 1990 to 2021. Abstracts of studies were then examined to see whether these studies analyzed the association between the polymorphism of each candidate gene and AUD and/or AUD-related conditions. The following criteria were necessary to be included in the review: (I) a peer-reviewed publication; (II) a case–control design; (III) independent from the others (i.e., duplications were discarded, and studies with previously published data were excluded); and (IV) sufficient information to compute an odds ratio (OR) and/or likelihood ratios (LR) along with 95% confidence intervals (CI). If two studies contained overlapping samples, the study with the larger sample size was kept for this meta-analysis.

### 2.2. Statistical Analysis

We first incorporated all the data from the selected studies to implement an overall analysis and then conducted stratified analyses. All these analyses were two-sided by default and were performed with the statistical programming language R. For this analysis, the Hardy–Weinberg equilibrium of each polymorphism in cases and controls was assessed. If available, the Pearson’s *χ*^2^ test or Fisher’s exact test was used for comparing gender, genotype, and allele distribution. The age difference was evaluated with the Pearson’s *χ*^2^ test and the Wilcoxon rank-sum test. To assess the association of each polymorphism with AUD and AUD-related conditions, the summary OR and their 95% CI were estimated for individual studies using the Pearson’s *χ*^2^ test if the contingency tables were available. Considering between-study heterogeneities caused by different genders, ethnicities, nationalities, diagnostic criteria, and so on, both fixed-effects and random-effects models were applied. For the fixed-effects analyses *(*i.e., the Mantel–Haenszel method), the effect size of each individual study was integrated using inverse variance methods to generate a pooled OR and 95% CI, assuming that the genotype effect is constant across studies and the detected variation is attributable to random variation; see Laird and Mosteller [22]. For the random-effects model, the effect sizes of individual studies were pooled, assuming that the heterogeneity among studies is attributable to both between- and within-study variations; see DerSimonian and Laird [23]. When compared to the fixed-effects model, the random-effects model is more conservative and produces a broad CI. Thus, when no significant heterogeneity is suspected among studies, the fixed-effects model tends to be more appropriate. Otherwise, the random-effects model is recommended. The significance of the pooled OR was ascertained using the standard *Z*-test (a 5% level of significance was adopted for all the analyses). The Bonferroni correction was applied if multiple comparisons had to be performed. Repetition of the meta-analysis was conducted to run a sensitivity analysis, with one study being omitted at a time, and no significant impact was reported. Furthermore, based on the values of pre-test odds = pre-test risk/(1-pre-test risk), the post-test odds were calculated as a product of OR and pre-test odds. Then, the post-test risk was estimated by the value of post-test odds divided by 1 + post-test odds. For the purpose of this analysis, the pre-test risk was assumed to be 8%.

Cochran’s *Q* and *I*2 tests were employed to evaluate the potential heterogeneity among various studies. The Cochran’s *Q* test was calculated as the weighted sum of squared differences between individual study effects and the pooled effect across studies, weighting the contribution of each study by its inverse variance. The *Q* statistic follows a *χ*^2^ distribution with *n* − 1 degrees of freedom (df), where *n* is the number of studies. The magnitude of *I*2 = 1 − df/*Q* then describes the proportion of variation across studies caused by heterogeneity rather than by chance; see Higgins and Thompson [24]. Evidence of heterogeneity between studies is indicated if *I*2 < 0.4. The method introduced by Hedges and Pigott [25,26] was used to approximate the statistical power of the meta-analysis. The funnel plot test of Begg and Berlin [27] and the regression test of Egger et al. [28] were performed to assess potential publication bias (see Galbraith [29]). The funnel plot utilizes a linear regression approach to measure the funnel asymmetry on the natural logarithm of the OR. The deviation of each study larger than the funnel-shaped distribution is predisposed to be more prominently asymmetric, indicating a possibility of publication bias. If the evidence of publication bias was noted, the trim and fill method of Duval and Tweedie [30] was attempted to adjust the meta-analysis results by imputing data from presumed missing studies. The total number of patients used in this analysis was 74,566 case–control subjects (Table 1).

## 3. Results

Table 2 summarizes the results of the statistical analyses, displaying the odds ratios (OR), 95% confidence intervals (CI) for OR, and a post-risk estimated population alcoholism prevalence of 8%. The dopamine receptor (DR) D1, DRD2, DRD3, DRD4, DAT1, COMT, OPRM1, and 5HTT all showed substantial risk favoring cases of AUD when compared to non-AUD controls using only the OR derived from this meta-analysis. However, because of insufficient sample sizes, similar significance was not obtained for GABRB3 and MAOA. The 95% CI supports this significant observation for OR except for GABRB3 and MAOA. Once again, this might be due to smaller sample sizes. Moreover, except for DRD1, COMT, GABRB3, and MAOA, the post-test risk was estimated at 8%. The pre-test risks all showed a positive gain, allowing for predictability of AUD-related conditions based on the polymorphism. However, we did find a positive gain with DRD2 (0.12), DRD3 (0.20), DRD4 (0.10), DAT1 (0.10), and 5HTTLPR (0.10). With additional samples, this could be overcome.

## 4. Discussion

We are not able to identify the OR for DRD1 currently. Given this, we will continue to obtain additional case–control studies for each allele in the GARS that will allow for the obtaining of OR statistics. It is anticipated that since we are close to having the proof for each allele showing risk for AUD over controls (albeit not RDS free as it should be), the incorporation of a new additional cohort of peer-reviewed and published works in the literature should enable a more robust finding.

In some cases, the risk estimates for one copy of each variant (not all due to the phenomena of heterosis) may be higher than ever for people with two copies of one variant. However, because a patient with either one or two copies is managed similarly in a clinical setting, the test report provided to the user will have the same interpretation as the test report for both genotypes. While some more work needs to be accomplished, it is important to highlight that at this stage, based on dichotomization of the GARS clinical data, any combination of these gene-associated alleles that reaches the level ≤ 4 loads onto the risk for drugs and gene-associated alleles that reaches the level ≤ 7 loads onto the risk for AUD [31]. Once this work is accomplished, it should provide unequivocal evidence for the validity of the selected risk gene-associated alleles. Although we claim that the selection of these candidate genes reflects dopamine dysregulation in the realm of hypodopaminergia, it is imperative to understand that the end function of dopamine at postsynaptic sites in the mesolimbic system is the net result of at least seven neurotransmitter system iterations. Dopamine is not alone and should not be considered in isolation.

With the advent of GWAS, there has been an explosion of very large studies related to the genetic polymorphic antecedents of AUD. While others have found evidence for a number of novel clusters of many genes, mostly second messengers, along with the requirement for convergence of these genes to candidates, our approach focuses on finite neurotransmitter pathways. We agree that future GWAS studies seem tantamount to unlocking additional candidates for AUD risk, but we believe the present approach has current hieratic value, requiring independent confirmation. While GWAS studies utilize very large sample sizes and many SNPs, we are not convinced that controls utilized in this sophisticated research reflect RDS-free symptomatology, which may prevent true associations between disease and the controls (disease-ridden).

## 5. Study Limitations

Due to differences among studies (e.g., gender, age, family history, ethnicities, nationalities, comorbidities, the severity of AUD conditions, or unscreened controls), considerable heterogeneity was observed, potentially resulting in publication bias (e.g., asymmetric funnel plots) and/or increased false-positive rates (e.g., Type-I error rates). Not all studies included in this analysis provided information regarding the aforementioned variables. Many of the studies did not provide such information to allow us to exclude or include patients with comorbid disorders in the reported samples; this makes it difficult to assess the effect of the above covariates inducing heterogeneity among studies. Even though the variables such as publication year, study populations, and diagnostic criteria did not seem to be potential sources of heterogeneity, other possible sources of heterogeneity, such as the onset and duration of alcohol addiction and other comorbid conditions or complications, could not be assessed in this meta-analysis. AUD is a complex addictive disorder with multifactorial pathogenesis and is often comorbid with other substance abuse or neuropsychiatric disorders, which likely share common genetic risk factors in the dopaminergic reward system. The potential interaction of candidate genes with others may also lead to heterogeneity among studies. The gene-by-gene or SNP-by-SNP interaction could not be examined due to insufficient studies on other variants that significantly contribute to the vulnerability of complex addictive phenotypes.

Furthermore, in this meta-analysis, only case–control studies were considered, which are more susceptible to sampling bias resulting from the potential differences between alcoholics and control groups when compared to family-based studies; see Gamma et al. [32]. The inter-rater reliability for selecting reported studies was not assessed, which might lead to some biases. Additionally, the control groups in most of the studies included in this review were not individuals randomly selected from the general population where cases were selected, contributing to the potential bias in the study results. Selection bias could also have occurred because the majority of the studies were published in English only. An improved meta-analysis should be conducted using more sophisticated analysis methods for controlling between-study heterogeneity and publication bias, as well as RDS-free controls.

Our work has been based on a number of candidate gene methods, which were first initiated by the work of Blum and Noble in 1990 [33] as the first confirmed candidate gene to be associated with alcoholism, as well as several other classic candidate gene association studies in terms of accepted methodology [34,35,36,37]. While we are cognizant of the pitfalls related to the candidate gene approach, including ancestry, we believe the candidate approach currently has a clinically relevant outcome and heuristic value. Certainly, the psychiatric genetic field is moving to GWAS instead of candidate gene research, but convergence to candidate genes is required to make real sense of the enormity of the data. One example of this type of GWAS analysis included a proxy-phenotype meta-analysis of Problematic Alcohol Use (PAU), which combined AUD and problematic drinking in 435,563 individuals of European ancestry [38]. They identified 29 independent risk variants, 19 of them novel. PAU was genetically correlated with 138 phenotypes, including substance use and psychiatric traits. Phenome-wide polygenic risk score analysis in an independent biobank sample (BioVU, N = 67,589) confirmed the genetic correlations between PAU and substance use and psychiatric disorders.

Moreover, a GWAS study involving a sample size of 1.2 million individuals involving both tobacco and alcoholism identified 566 polymorphisms in 406 loci associated with various stages of tobacco use (heaviness, cessation, initiation) and alcohol use, with 150 loci providing evidence of a pleiotropic association [39]. However, when convergence was applied, the authors identified several genes involved in dopaminergic, nicotinic, and glutamatergic neurotransmission as well as other systems. Our concern related to these GWAS studies and our subsequent evaluation is that the controls, for the most part, that are utilized have not been adequately screened to eliminate all reward deficiency symptomatology and associated disorders (i.e., gambling, hoarding, obesity, ADHD, etc.).

Hundreds of published studies about dopamine and its role in addictive and compulsive behaviors were reviewed in search of a reliable gene-based diagnostic test to identify heritable risk factors for RDS. Our goal was to illustrate the process used to select the risk alleles of reward genes for the GARS test and to address the limitations caused by inconsistent results in many case–control behavioral association studies. Limitations are perhaps due to the failure of investigators to adequately screen controls for drugs, AUD, and RDS behaviors, including nicotine dependence, obesity, pathological gambling, and internet gaming addiction. One example of accomplishing RDS-free controls is derived from Blum et al. [40], which revealed the prevalence of the DRD2 A1 allele in unscreened controls (33.3%) compared to “Super-Controls” (highly screened RDS controls (3.3%) in proband and family). Therefore, to provide the best possible statistical analysis, any RDS-related behaviors must be eliminated from the control group to avoid comparing the phenotype to disease-ridden controls.

In conclusion, unlike one gene-one disease (OGOD), RDS is polygenetic and complex. Even though the genes evaluated are not the only ones associated with AUD, we focused on these genes and the related polymorphisms because the available GARS test only assesses genes chosen based on hypodopaminergia. Therefore, other genes such as alcohol metabolism genes (e.g., alcohol dehydrogenase) combined with GARS may provide an even stronger association in terms of risk. Certainly, utilizing genetic risk assessment in any form will help identify early on the future risk, now termed “preaddiction” [41], which is only one part of the equation to help attenuate future AUD or other RDS behaviors. Early identification is indeed important in terms of prophylaxis, especially in our youth, as evidenced by common brain mapping of addiction [42].

### Future Perspective

From 1999–2019, nearly 500,000 people have died from an opioid overdose, involving any opioid, including prescription and illicit opioids [43]. While we are encouraging the short-term utilization of opioid substitution treatment (OST), which may be useful for OST in the short-term, to reduce harm, it is important to continue the quest for therapeutically superior options. The goal for the future is to find a much better option.

To that end, we suggest one important way to help reduce overdose rates would be OST incorporated into preventive and therapeutic strategies of metabolic assessments in conjunction with established genetic risk assessment testing, such as the Genetic Addiction Risk Severity (GARS) complemented by, in combination with RDS, Questionnaire RDSQ29 [44], or any other viable genetic assessment tool, to provide the potential early identification of a newly identified concept, what has been now termed “Preaddiction”, similar to “Prediabetes”, and assist in categorizing people with a high vs. low likelihood of risk for glucoregulatory abnormalities, RDS and its associated bioinformatics (all addictions), which would contribute to a lesser likelihood of risk prior to deciding on the prescribing of long-term powerful OST. Additionally, in the future, based on required research, consideration must be given to the “Standard of Care”, which may be adjusted as the putative induction of “homeo-dopamine hedonoomeostasis” is gaining further support in clinical and basic research, utilizing a number of pathophysiology-driven proven modalities, including repetitive Transcranial Magnetic Stimulation (TMS), DBS (Deep Brain Stimulation), and pro-dopamine regulation (e.g., KB220), as well as other viable solutions.

## 6. Conclusions

**Figure 2:**
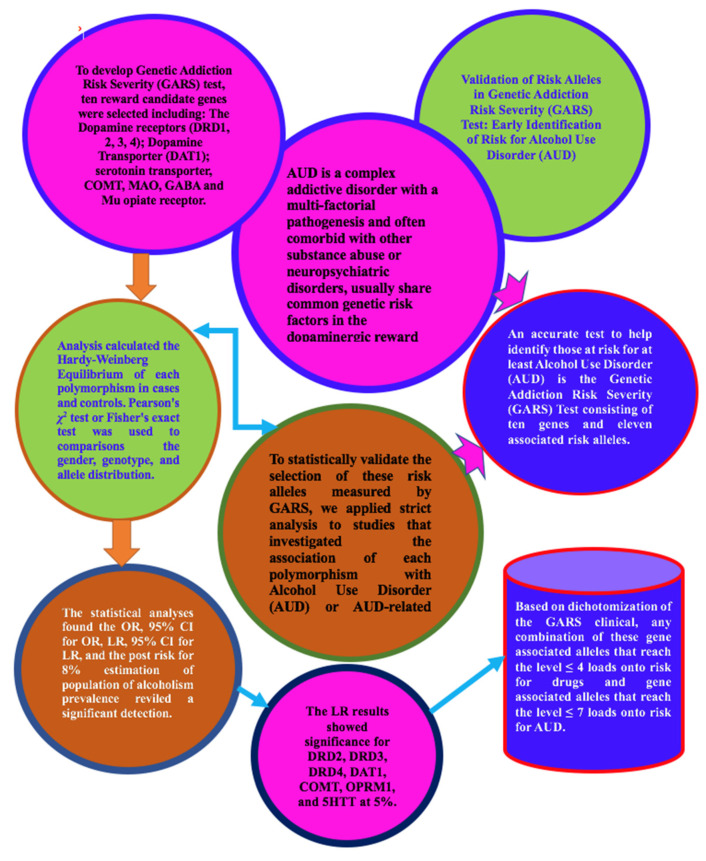
Schematic of statistical validation of risk alleles in genetic addiction risk severity (GARS) test: Early identification of risk for Alcohol Use Disorder (AUD) in 74,566 case-control subjects.

In summary, the results of the statistical analyses displayed the OR, 95% CI for OR, and a post-risk estimated population alcoholism prevalence of 8%. Using only the OR derived from this meta-analysis of 74,566 case–control subjects, DRD2, DRD3, DRD4, DAT1, COMT, OPRM1, and 5HTT all showed significant risk favoring cases of AUD compared to non-AUD controls. While the DRD1 gene did show a significant OR, the small sample size prevented us from capturing the post-risk for 8% of the population. Figure 2 represents a schematic of our model.

## Figures and Tables

**Table 1:** Gene polymorphisms under consideration and the literature summary.

Gene/Polymorphism	Number of Reference Sources	95% CI Overall Summary
Dopamine D1 Receptor (DRD1): rs4532—risk allele G	3	The genetic variation in DRD1 and its relationship to a predisposition to alcoholism have been supported by various studies. A statistically significant association of DRD1 rs4532 polymorphism with alcohol dependence was found among Indian males (90 cases vs. 122 controls). Other studies also demonstrated that this could be associated with the impulsivity and aggressiveness of AUD patients.
Dopamine D2 Receptor (DRD2): rs1800497—risk allele A1	118	From the meta-analysis of numerous case–control studies (total of 18,290 cases vs. 19,809 controls, including US Caucasian, native and African-American, British, French, Italian, Swedish, Finnish, Spanish, Mexican, Brazilian, Scandinavian, Japanese) pooled with the random effect models, the DRD2 rs1800497 was found to be associated with a risk of AUD and several AUD-related conditions.
Dopamine D3 Receptor (DRD3): rs6280—risk allele C (Ser9Gly)	3	Several case–control studies investigated the association between the DRD3 rs6280 polymorphism and alcohol dependence. In a Korean study (243 cases vs. 130 controls), the DRD3 rs6280 polymorphism was significantly associated with AUD development.
Dopamine D4 Receptor (DRD4): rs1800955—risk allele C (48bp repeat VNTR)	35	A meta-analysis of various case–control studies (total 2997 cases vs. 2588 controls, including US Caucasian, Mexican-American, Indian) pooled with the random effect models found that the DRD4 rs1800955 polymorphism was associated with the risk of developing AUD and AUD-related conditions.
Dopamine Transporter Receptor (DAT1): SLC6A3 3′-UTR—risk allele A9 (40bp repeat VNTR)	43	The central dopaminergic reward pathway is likely involved in alcohol intake and the progression of alcohol dependence. DAT1 is a primary mediator of dopaminergic neurotransmission. From the meta-analysis of numerous case–control studies (total 3790 cases vs. 3446 controls) pooled with the random effect models, the DAT1 SLC6A3 3′-UTR risk allele was found to be marginally associated with a risk of AUD and/AUD-related conditions.
Catechol-O-Methyltransferase (COMT): rs4680—risk allele G (Val158Met)	13	A plethora of evidence supports COMT as a candidate gene that likely contributes to schizophrenia and substance use disorder. A meta-analysis of several case–control studies (total of 1212 cases vs. 933 controls, including US Caucasian, Finnish, Croatian, and Taiwanese) pooled with a random effect model, the association of COMPT rs4680 polymorphism with the risk of AUD and AUD-related conditions was found to have marginal statistical significance.
µ-Opioid Receptor (OPRM1): rs1799971—risk allele G (A118G)	28	Opioid receptors play an essential role in ethanol reinforcement and alcohol dependence risk. Some features of alcohol dependence are likely associated with polymorphisms of the OPRM1 gene expressing µ-opioid receptors. From the meta-analysis of case–control studies (total of 3096 cases vs. 2896 controls, including US Caucasian, Spanish, Turkish, and Asian) pooled with the random effect model, the results indicated that the association of a functional OPRM variant and the risk of alcohol dependence was found to have marginal statistical significance.
γ-Aminobutyric Acid (GABA) A Receptor, β-3 Subunit (GABRB3): CA repeat—risk allele 181	6	The GABAergic system has been implicated in alcohol-related behaviors. From case–control studies (171 cases vs. 45 controls), the association of variants of the GABRB3 gene with alcohol dependence is, however, inconclusive. A more extensive controlled study is required for improved results.
Monoamine Oxidase A (MAO-A): 3′ 30bp VNTR -risk allele 4R DNRP	6	The function of monoamine oxidase (MAO) in alcoholism was determined using several case–control studies (170 cases vs. 177 controls). Although genetic heterogeneity is suspected of underlying alcoholism and MAO-A mutations may play a role in susceptibility to alcoholism, the overall results were not found to be statistically significant. A more extensive controlled study is required to obtain conclusive results.
Serotonin Transporter Receptor (5HTT) Linked Promoter Region (5HTTLPR) in SLC6A4: rs25531—risk allele S′	20	Serotonin (5-HT) has been demonstrated to regulate alcohol consumption. Since the activity of the 5-HT transporter protein (5-HTT) regulates 5-HT levels, it may contribute to the risk of alcohol dependence. From the meta-analysis of some case–control studies (total 9996 cases vs. 9950 controls) pooled with the random effect models, the association between alcohol dependence and a polymorphism in the 5-HTTLPR was significant.

**Table 2:** Odds ratios and likelihood ratios of polymorphisms under consideration.

Gene/Polymorphism	OR	95% CI for OR	Post Risk
Dopamine D1 Receptor (DRD1): rs4532—risk allele G *	1.77	(1.01, 3.10)	-
Dopamine D2 Receptor (DRD2): rs1800497—risk allele A1	1.45	(1.15, 1.90)	0.12
Dopamine D3 Receptor (DRD3): rs6280—risk allele C (Ser9Gly)	3.37	(1.54, 7.40)	0.20
Dopamine D4 Receptor (DRD4): rs1800955—risk allele C (48bp repeat VNTR)	1.56	(1.04, 2.36)	0.10
Dopamine Transporter Receptor (DAT1): SLC6A3 3′-UTR—risk allele A9 (40bp repeat VNTR)	1.18	(1.00, 1.45)	0.10
Catechol-O-Methyltransferase (COMT): rs4680—risk allele G (Val158Met)	1.43	(0.98, 2.10)	0.083
µ-Opioid Receptor (OPRM1): rs1799971—risk allele G (A118G)	1.47	(1.00, 2.18)	0.13
γ-Aminobutyric Acid (GABA) A Receptor, -3 Subunit (GABRB3): CA repeat—risk allele 181	0.33	(0.14, 0.79)	0.06
Monoamine Oxidase A (MAO-A): 3′ 30bp VNTR-risk allele 4R DNRP	0.62	(0.15, 2.63)	0.05
Serotonin Transporter Receptor (5HTT) Linked Promoter Region (5HTTLPR) in SLC6A4: rs25531—risk allele S′	1.23	(1.07, 1.40)	0.10

* Not enough data. It is noteworthy to point out that for each gene polymorphism, the number of cases and controls has been indicated in Table 1.

## Data Availability

Not applicable.
